# Significance of Serum Lactate Dehydrogenase as a Prognostic Marker and Outcome Predictor in Patients With Breast Cancer

**DOI:** 10.7759/cureus.55932

**Published:** 2024-03-11

**Authors:** Geetika Malhotra, Rajesh G Gattani, Raju K Shinde, Sanjeev G Gianchandani, Krushank Nayak, Ankur Salwan

**Affiliations:** 1 General Surgery, Jawaharlal Nehru Medical College, Datta Meghe Institute of Medical Sciences, Wardha, IND; 2 Minimal Access and Robotic Surgery, Anglia Ruskin University, Chelmsford, GBR; 3 Orthopaedic Surgery, Jawaharlal Nehru Medical College, Datta Meghe Institute of Medical Sciences, Wardha, IND

**Keywords:** ca breast prognostic markers, lactate dehydrogenase, tnm stage, lymphovascular invasion, carcinoma breast

## Abstract

Background

Breast carcinoma has been the most prevalent cancer in women, with research-based evidence showing a significant rise in the incidence of cancer and related morbidity and mortality in the Indian subcontinent. The predictive value of plasmatic lactate dehydrogenase (LDH) levels has been studied in breast cancer. Numerous studies have connected high LDH values to a poor prognosis, increased risk of incidence, recurrence, and associated mortality in patients with breast carcinoma. This study aimed to assess the clinical profile of breast carcinoma and determine the correlation of serum lactate dehydrogenase levels with the stage of the disease and assessment of high-risk features using histopathology and immunohistochemistry.

Methods

A total of 75 patients with carcinoma breast were enrolled for this study and classified into two groups: upfront surgery and post-adjuvant therapy. Serum LDH levels were estimated a day before the surgery (baseline) and on postoperative days 1, 7, 14, and 30. The clinical tumor, node, metastasis (cTNM) staging was correlated with pathological tumor, node, metastasis TNM (pTNM) staging and immunohistochemistry findings.

Results

The clinical characteristics of breast cancer, serum LDH levels, and stage of the disease were collected and analyzed. A significant decreasing trend was noted in LDH values post-op days, and statistically significant higher LDH values were noted in the triple-negative group, positive lymph nodes, and positive lymphovascular invasion patients.

Conclusion

Regularly elevated levels or an unanticipated rise in serum LDH might indicate poor outcomes. Hence, this non-specific enzyme marker can be suggested to be used routinely to assess disease outcomes.

## Introduction

Breast carcinoma is a commonly observed cancer type in females and is reported to have a prevalence of 124 per million [1]. India has a lower prevalence of breast cancer than the West; however, it is becoming more common among urban Indian women. According to Indian Council of Medical Research (ICMR) data statistics, breast cancer contributes to more than 30% of all cancers. ICMR, based on a recent study, also reported an increased incidence of breast cancer cases from 1,06,124 in 2015 to 1,23,634 in 2020 [2]. Based on current research evidence, an increased rate of cancer-related mortality, morbidities, and disease presentation has been noted in the Indian subcontinent [3]. Though the survival time (medial) for breast cancer is approximately 34 months and has improved despite new therapies and standards of care, the illness is still incurable [4]. Prognostic outcomes, either favorable or adverse, can be better predicted based on the assessments of clinical, histological, and radiological presentations at different disease stages. Mainly, breast lumps are assessed by self-examination, radiological screening, and histopathology of the tissue using fine needle aspiration cytology. Although histopathological screening can provide the confirmational diagnosis of carcinoma breast diagnosis, it can be a burden for developing countries like India, where there are resource limitations. Newer research has suggested circulating tumor cells, inflammatory factors, exosomes, and circulating tumor DNA as novel prognostic biomarkers for breast cancer [5]. Insights on tumor presentations can also be gained from various biomarkers, such as enzyme levels and blood cell ratios, which can be utilized for developing prognostic predictors [6].

Clinical care for this malignancy is based on several prognostic factors, most notably the lymph node stage, tumor size, and histological grade. Independent investigations have shown that various additional traits such as Clinical T, Clinical nodal status, pathological staging, pathological grade, Bloom-Richardson (BR) score, lymphovascular invasion, margin, and immunohistochemistry like triple-negative status and positive LN are relevant for prognosis. Therefore, efforts have been made to incorporate these traits into the practical indices. Breast, gynecological, and colon cancer risks have been associated with elevated serum lactate dehydrogenase (LDH) levels. Research studies on metastatic breast cancers reported a relationship between increased LDH levels in blood and poor outcomes such as recurrence and mortality [7]. LDH is a crucial enzyme in the glycolysis cycle. In lack of oxygen, pyruvate is formed from glucose. This reaction is sped up by the LDH enzyme. These anaerobic conditions are observed in the tumor environment due to extreme hypoxia. Due to a lack of oxygen, the LDH-A gene is upregulated by the over-expression of hypoxia-inducible factor 1 transcription factor [8,9]. It is also noted that cancer cells are reported to use anaerobic pathways for energy generation, regardless of sufficient oxygen availability. Serum total LDH levels and the gene for LDH-A, an isoenzyme, are frequently elevated in patients with cancer. Numerous studies have connected these characteristics to poor prognosis [10]. The use of serum LDH levels as a predictive marker for breast cancer has been well-proven in terms of anticipating treatment outcomes and mortality [11]. However, there are different schools of thought on this, as there is mixed research evidence on the association between serum LDH levels and breast cancer [12]. Hence, we aim to study the correlation of histological traits, disease stage, and progression with serum LDH in the study participants.

## Materials and methods

This prospective study enrolled 75 breast cancer patients confirmed by histological analysis at a tertiary care center in Central India from December 2020 to December 2022. Newly diagnosed patients planned for surgical management for carcinoma breast and patients who were scheduled for surgical management after neo-adjuvant chemotherapy were enrolled in the study. Patients who recently had mastectomy/ lumpectomy, liver disease, myocardial infarction, polycythemia, megaloblastic anemia, hemolytic anemia, rheumatic fever, and other severe comorbidities, were inoperable, had recurrent/ bilateral carcinoma breast and males with carcinoma breast were excluded. Patients who were noncompliant with the standard treatment protocol for carcinoma breast and unwilling to participate were also excluded. The patients clinically diagnosed with carcinoma breast were categorized as per inclusion and exclusion criteria, and breast lump/s were examined thoroughly. Clinical tumor, node, metastasis (cTNM) staging was followed by radiological and histological confirmation and associated risk analysis. All the cases were discussed for further management by the tumor board. The patients were categorized into two groups: undergoing upfront surgery (adjuvant therapy) and neoadjuvant therapy (chemotherapy followed by surgery). Patients who underwent neoadjuvant chemotherapy were included in the study population once there was a downstaging of the disease. Serum LDH levels were estimated at baseline (a day before the surgery) and serial estimations at postoperative day (POD) 1, 7, 14, and 30. The cTNM staging was correlated with pathological tumor, node, metastasis (pTNM) staging, and immunohistochemistry findings. Baseline and postoperative serum LDH levels were associated with clinical staging, pathological staging, histopathology, immunohistochemistry findings, and correlation with disease stage.

Statistical analysis

SPSS version 24.0 (IBM Corp., Armonk, USA) was used to analyze the data with version 7.0 of GraphPad Prism software (GraphPad, San Diego, USA). Descriptive and inferential statistics were carried out using the Chi-square test with p<0.05 read as significant.

## Results

A total of 75 patients aged 21 - 90 years were enrolled for this study. Details of the same have been presented in Table 1.

**Table 1:** Age-wise distribution of the study participants

Age (in years)	N (%)
21-30	7 (9.30)
31-40	12 (16.00)
41-50	25 (33.30)
51-60	17 (22.70)
61-70	10 (13.30)
71-80	2 (2.70)
81-90	2 (2.70)

The lump was observed in the right breast in 33 (44%) patients, and the rest of the patients (42, 56%) had a lump in the left breast. Most participants had no comorbidities (88.0%), with hypertension observed in 9.3%, bronchial asthma in 1.3%, and diabetes and hypertension in 1.3% of participants with a mean hospital stay of 21.13±11.18 days. The majority of the study participants were presented for upfront surgery. The patient’s disease profile based on varied clinical screening parameters is shown in Table 2.

**Table 2:** Patient disease profile and management HER-2: human epidermal growth factor receptor 2

-	Parameter	N	%
Immunohistochemistry screening	Estrogen (+)	37	49.3
	Progesterone (+)	25	33.3
	HER-2 (+)	42	56
Pathological Diagnosis	Infiltrating Ductal Carcinoma (NOS)	75	100
	Invasive Lobular Carcinoma (NOS)	0	0
	Others	0	0
Case Management	Upfront surgery	62	82.7
	Post-Neoadjuvant	13	17.3

All of the patients were diagnosed with infiltrating ductal carcinoma. Tumor size staging (cT and pT) and nodal staging (cN and pN) have been listed in Table 3.

**Table 3:** Comparative analysis of clinical and pathological analyses on disease stages cT: clinical tumor; pT: pathological tumor; cN: clinical node; pN: pathological node

Tumor size	cT Staging (n/%)	pT Staging (n/%)
T1	6 (8.0)	6 (8.0)
T2	38 (50.7)	38 (50.7)
T3	22 (29.3)	21 (28.0)
T4	9 (12.0)	10 (13.3)
Nodal Staging	cN Staging (n/%)	pN Staging (n/%)
N0	36 (48.0)	39 (52.0)
N1	22 (29.3)	17 (22.7)
N2	17 (22.7)	11 (14.7)
N3	0 (0)	8 (10.7)

A Bloom Richardson (BR) score of 6 or 7 was observed majorly in 43 (57.3%) study participants. Nottingham combined histologic grade/BR grade revealed maximum participants in grade II (44 (58.7%)). The difference in the mean values of LDH at the baseline was found to be non-significant with respect to BR scoring levels, pathological histopathology evaluation (HPE) grades, histopathological HPE grades, and different clinical stages are indicated in Table 4.

**Table 4:** Change in lactate dehydrogenase (LDH) levels over time in the study group BR: Bloom Richardson Grade; HPE: Histopathology evaluation; CS: Clinical stage; U: Unit; L: Liter

-	Parameter	LDH (Baseline) Mean ± SD	P value
BR Score	BR 4	433.00 ± 133.50	0.603
	BR 5	297.78 ± 156.08	
	BR 6	305.36 ± 97.61	
	BR 7	312.76 ± 115.80	
	BR 8	320.90 ± 156.37	
	BR 9	316.10 ± 113.10	
Pathological	HPE I	281.33 ± 43.10	0.512
	HPE II	299.00 ± 107.75	
	HPE III	339.74 ± 139.04	
Histopathological	HPE I	330.38 ± 150.57	0.981
	HPE II	306.20 ± 105.17	
	HPE III	325.95 ± 138.19	
Clinical Stage	CS I	248.00 ± 0.0	0.276
	CS II	295.48 ± 102.75	
	CS III	349.82 ± 142.71	

Assessment for the change in LDH over time in breast cancer patients undergoing upfront resection and post-neoadjuvant chemotherapy showed initially increased post-operative LDH levels before falling to normal levels (Table 5).

**Table 5:** Association between type of case and lactate dehydrogenase (LDH) (Baseline)

LDH (Baseline)	Upfront resection	Post-Neoadjuvant case type	Wilcoxon-Mann-Whitney U Test	p-value
Mean ± SD (Min – Max)	302.55 ± 114.11 (154 – 663)	375.15 ± 138.45 (209 – 666)	261.000	0.048

Changes in LDH values from baseline and different postoperative days (POD) 1, 7, 14, and 30 days were found to be as mentioned in Table 6.

**Table 6:** LDH levels at baseline and different postoperative time periods LDH: Lactate dehydrogenase; POD: Postoperative day; U/L: Unit per litre

Time period	LDH (U/L)(Mean ± SD)	Range	Chi-square	p-value
Baseline	315.13 ± 120.86	154.00 - 666.00	257.6	<0.001
POD 1	300.92 ± 113.94	144.00 - 620.00		
POD 7	291.27 ± 112.19	132.00 - 600.00		
POD 14	271.73 ± 111.57	101.00 - 590.00		
POD 30	256.35 ± 112.80	101.00 - 623.00		

The association between histopathology evaluation (HPE), lymphovascular invasion, and LDH was also significant, with the highest LDH levels in the positive lymphovascular invasion group (Table 7).

**Table 7:** Association Between LVI and LDH HPE: Histopathology evaluation; LVI: Lymphovascular invasion; POD: Postoperative day

LDH	HPE: LVI Positive (Mean ± SD)	HPE: LVI Negative (Mean ± SD)	p-value (inter-group comparison)
Baseline	343.95 ± 128.35	274.23 ± 97.32	0.010
POD 1	331.55 ± 117.95	257.45 ± 93.55	0.004
POD 7	318.48 ± 115.15	252.65 ± 97.01	0.006
POD 14	300.91 ± 115.21	230.32 ± 93.07	0.002
POD 30	287.39 ± 119.24	212.29 ± 87.10	0.001
p-Value (intra-group comparison)	<0.001	<0.001	-

**Figure 1:**
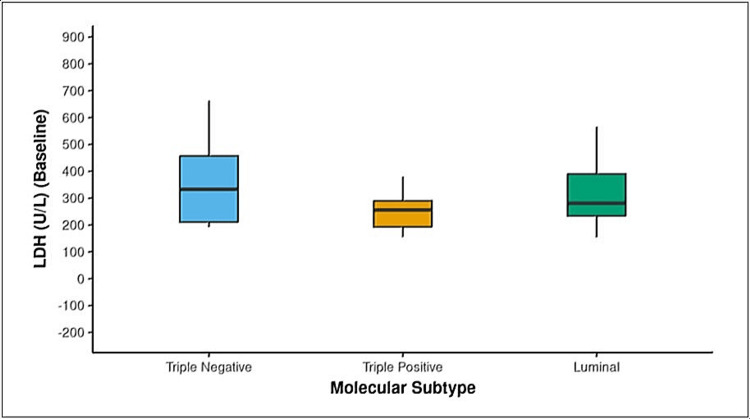
Association between molecular subtype and lactate dehydrogenase (LDH) (Baseline) U/L: units per litre

The clinical stage I group had a substantial difference in LDH levels. The mean serum LDH levels increased significantly between stages I and III, but this trend was not statistically significant. The triple negative group molecular subtype had a significant difference with the highest mean LDH value (Table 8) (Figure 1).

**Table 8:** Association between molecular subtype and serum lactate dehydrogenase (LDH) levels

LDH (Baseline)	Molecular Subtype			Kruskal Wallis Test	
	Triple Negative	Triple Positive	others	χ2	p-value
Mean ± SD	364.71 ± 139.58	250.69 ± 69.67	315.02 ± 118.25	6.177	0.046
Min – Max	192 - 663	155 – 380	154 – 666		

**Figure 2:**
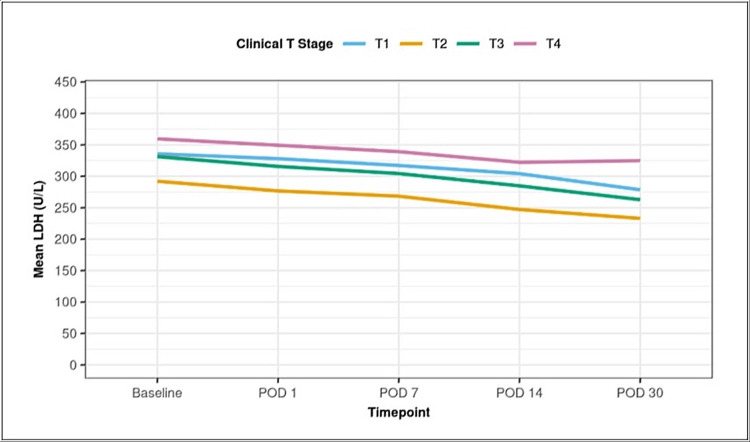
Comparison of the clinical T-stage with lactate dehydrogenase (LDH) levels over time U/L: units per litre

A Kruskal-Wallis significance test was used for inter-group comparisons, and the Friedman test compared intra-group data. The generalized estimating equations method was used to explore the difference in change in LDH between the groups over time, which was found significant by p<0.001. A significant difference in LDH values was observed between the groups based on lymphovascular invasion, with higher LDH in the patient subset with the presence of lymphovascular invasion. Intragroup comparisons were analyzed by the Friedman Test, and over time, overall values were tested by generalized estimating equations. The difference was observed at the following time points: Baseline, POD 1, POD 7, POD 14, and POD 30. Higher levels of serum LDH in these patients might be a warning alarm for poor outcomes, as presented in (Table 9) and (Figure 2).

**Table 9:** Comparative analysis of clinical T stage in terms of change in LDH over time POD: Postoperative, LDH: Lactatedehydrogenase

LDH (U/L)	Clinical T Stage (Mean ± SD)				Kruskal Wallis Test p-value
-	T1	T2	T3	T4	-
Baseline	335.67 ± 65.77	292.05 ± 118.18	331.23 ± 124.92	359.56 ± 145.19	0.219
POD 1	328.00 ± 68.19	276.66 ± 114.33	315.64 ± 109.24	349.33 ± 137.14	0.094
POD 7	317.17 ± 69.80	268.29 ± 111.97	304.32 ± 111.25	339.11 ± 129.77	0.138
POD 14	304.17 ± 61.40	247.13 ± 108.53	284.73 ± 111.59	322.22 ± 136.47	0.087
POD 30	278.50 ± 60.23	232.95 ± 110.38	262.73 ± 107.19	324.78 ± 143.41	0.053
P value	<0.001	<0.001	<0.001	<0.001	-
Intergroup LDH values (Overall time)	<0.001				

## Discussion

Numerous researchers have attempted to assess the function of biochemical indicators in the diagnosis and prognosis of breast cancer. However, these markers have not yet shown any promise regarding diagnostic criteria. The unregulated growth of cells causes a change in the quantities of biomolecules such as proteins, hormones, and enzymes in both blood and the affected tissue, which is reflected by tumor-associated indicators. Therefore, these changes in serum enzymatic levels can be counted as a reasonable marker of malignancy in early disease stages, where remarkable sensitivity and specificity parameters are available. Numerous researches have looked into the serum LDH level’s predictive relevance in individuals with breast cancer [13]. Most participants in this research study (49.5%) were between the ages of 31 and 50. Research studies have reported that the average age of patients is between 31 and 60 years [14-17]. Breast cancer’s laterality is a critical concept in Western literature, which contends that left-sided breast cancer is more prevalent due to handedness and breast hemispheric laterality. We observed left-sided breast carcinoma in 56% of patients in our study. Similar findings were reported by other researchers, with left breast cancer more common in women than right [18,19]. 

Clinical T stage

Shet *et al*., reported T2 as the most prevalent T-stage at a presentation seen in 56.32% and 64.64% of young and older breast cancer patients, respectively [20], supported by Wang et al., which reported T2 tumors in 35.68% of patients under the age of 40 as compared to 37.93% patients greater than 40 years as the most prevalent presentation [21]. 

Clinical lymph nodal stage

Rathod *et al*. found that the mean lymph node ratio in this group of patients was 0.42, which was consistent in this study, with N0 as the most frequent nodal stage followed by N1, N2, and N3 [22]. 

Clinical staging

Clinical staging in the study group was consistent with the research literature categorizing most patients in clinical stages II or III [22,23]. According to Khokher *et al*., Stages II, III, and IV had stage distributions of 32%, 35%, and 23%, respectively, which did not match the results of our study [24]. However, in a study of breast cancer cases presenting at two cancer hospitals in Lahore, 63% and 71% of patients presented at advanced stages (TNM Stages III and IV) [25].

Pathological diagnosis

As per our observations, all patients had ductal cancer, with the majority having infiltrating ductal carcinoma. This is in accordance with other scientific publications reporting ductally invasive tumors as the most prevalent histopathologic type, followed by other histopathologies such as medullary carcinoma and lobular carcinoma [20,22,26,27].

Type of case

In most cases, neoadjuvant chemotherapy successfully downgraded the tumors’ stage; nevertheless, the study's complete clinical response rate was lower. This is in accordance with the research conducted by Shrivastava *et al*., where 70% underwent surgical treatment [23], with 4.0% of patients who experienced a significant reduction in tumor size, neoadjuvant chemotherapy was administered. Shenkier *et a*l., revealed improved outcomes when chemotherapy was administered before surgery [28].

Pathological staging

Patients classified into pT stage II and stage III were observed as 50.7% and 28.0%, respectively, in similarity with other research by Rathod et al., and Samanta et al., with pT stage 2 as prevalently observed in their patients [22,29].

Assessment of change in LDH over time

A statistically significant decline was similar to Agrawal *et al*., which demonstrated an initial rise of LDH levels on POD-7 before falling to normal levels [14]. Though a few research studies have presented data with no statistical difference in serum LDH levels between baseline and POD 7, post-treatment serum LDH levels at one month were connected with treatment response, similar to this study [30].

Association between HPE, LVI, and LDH

There was a considerable difference in LDH between the two groups, with the median LDH being highest in the group with positive lymphovascular invasion (LVI). Gurleyik *et al*., considered that cancer cells present within a specific endothelial-lined area (either lymphatic vessels or blood vessels) in the breast are definitive of lymphovascular invasion [31]. LVI has been linked in certain studies to a higher risk of axillary nodal metastases, distant metastases, and death. Others, however, have demonstrated that it is not a reliable predictor of overall survival. Liao et al. have shown that LVI is a standalone factor associated with poor prognosis in breast cancer patients [32]. When found morphologically in the peri-tumoral region, LVI is a sign of possible metastasis and is closely linked to a poor prognosis in various solid tumors, including breast cancer.

Association between HPE, pathological stage, and LDH

There was no appreciable variation between the groups (T-stage) regarding LDH. There was also no noticeable LDH variation across the groups (N-stage). Though not statistically significant, serum LDH levels increased sequentially as the illness stage increased, which was noticed majorly in stage I and stage III comparisons. In the study by Mohammed Saheb et al., a stage-to-stage comparison revealed a non-significant rise between stages I and II. However, a substantial increase in serum LDH activity was seen between stages II and IV [33]. The present study findings are in accordance with the Basnyat AS et al. study, which reported a significant increase in serum LDH levels with worsening disease severity (stage) [34]. A research study by Agarwal et al., reported higher serum LDH levels in breast cancer patients than in the control group, with a correlation observed in the clinical stage and size of the tumor, which is consistent with the findings of this research study [14]. Another research mentioned that the illness stage was associated with higher serum levels of lactate dehydrogenase [35].

Association between type of case and LDH

There was a substantial difference in LDH between the two groups, with the post-neoadjuvant group having the highest median LDH. This is in agreement with the study conducted by Dennison *et al*., in which an LDH level of 252.0 ng/L was found to be a reliable indicator of complete remission following neoadjuvant chemotherapy. Independent of conventional prognostic indicators, expression of LDHB predicted response to neoadjuvant chemotherapy within clinical subgroups. The findings support the prospective clinical assessment of LDH as a response predictor for breast cancer patients undergoing neoadjuvant chemotherapy [36]. Masood presented that high LDH-B predicted pathologic complete response to neoadjuvant treatment for both hormone receptor (HR)-positive/human epidermal growth factor receptor 2 (HER2)-negative and triple-negative tumors (OR 14 4.1, p<0.001) [37].

Association between clinical stage and LDH

Within the groups (T Stage), there was a noticeable decline in serum LDH. Regarding LDH, there was an appreciable difference between the groups (N Stage), but it was statistically insignificant. The mean serum LDH levels increased significantly between stage I (248.0) and stage III (349.82), but this trend was not observed to be statistically significant. The observations of this study were in agreement with the studies of Agrawal et al., where it was found that the patients with higher stages had greater LDH levels than those with lower stages [14]. However, Mehdi et al. concluded that patients in higher clinical stages had higher LDH activity than those in lower stages [38].

Association between molecular subtype and LDH

The median LDH was highest in the triple-negative group molecular subtype, with a significant difference in the LDH levels among all three groups. This is further supported by the study conducted by Dong et al., where triple-negative breast cancer was strongly correlated with serum LDH status, tumor LDH-A expression, and slope of serum LDH status [39]. McCleland *et al*. found that in contrast to benign and luminal breast cancers, LDH-B is strongly related to triple-negative breast cancer, in consistency with these results [40].

These cancerous cells’ anaerobic glycolysis and ability to meet their metabolic needs are both aided by the elevated LDH level. Individuals with benign breast disease, such as fibroadenomas, have LDH values near to carcinoma (30860 IU/l), however, patients with breast malignancy have serum LDH values that are specific and also correlate with clinical TNM staging [41]. LDH is also recognized as a marker for myocardial infarction, hemolysis, inflammation, and tissue injury. The prognostic usefulness of elevated LDH levels has been demonstrated in many cancers, including germ cell tumors, lymphoma, melanoma, and renal cell carcinoma [42]. While more recent studies have suggested that LDH activity can work as an all-encompassing predictor of the prognosis for cancer. Because of the increased lactic acid levels and LDH enzyme activity, a sizeable number of malignant cells that are actively growing and whose metabolic feature is anaerobic glycolysis are harmed by serum LDH. This increase in serum LDH levels may be the reason for the tumor cells' massive excretion of lactic acid since the anaerobic glycolytic pathway functions differently in tumor cells than in normal cells [43]. Concluding, we observed a significant relationship between post-surgery LDH levels at various time intervals which was irrespective of the stage of the disease in patients with breast carcinoma. Based on these results, we recommend the use of post-surgery LDH levels in prognostic prediction in different disease groups. There are a few limitations of this study, which can be mentioned as small sample size, small follow-up duration, same ethnicity, and single-center study. The results of this study can be further validated by longer duration follow-up, including more number of participants with diversified cultural backgrounds.

## Conclusions

Biochemical markers have shown promising results in the diagnosis of breast cancer. However, their use as diagnostic criteria has not been beneficial. Further research can be favorable to develop better insights into the utilization of these biochemical indicators in the diagnosis and treatment of breast cancer. Researchers have explored the use of biochemical indicators in diagnosing breast cancer, but their effectiveness as diagnostic criteria has not been proven. Serum LDH level tracking might be utilized to correlate the therapeutic outcomes of these patients. Higher serum LDH levels can be associated with a greater degree of lymphovascular invasion in the triple-negative breast cancer patient group. Serum LDH levels that are consistently high or abruptly rise post-surgery or over an extended period might be indicative of poor outcomes. Hence, this non-specific enzyme marker can be routinely used for assessing disease outcomes.
